# Heat shock protein 27 (HSP27): biomarker of disease and therapeutic target

**DOI:** 10.1186/1755-1536-5-7

**Published:** 2012-05-07

**Authors:** Aparna Vidyasagar, Nancy A Wilson, Arjang Djamali

**Affiliations:** 1 Nephrology Division, Department of Medicine, University of Wisconsin, H4/564 CSC, 600 Highland Avenue, Madison, WI, 53792, USA

## Abstract

Heat shock protein 27 (HSP27) is a multidimensional protein which acts as a protein chaperone and an antioxidant and plays a role in the inhibition of apoptosis and actin cytoskeletal remodeling. In each of these capacities, HSP27 has been implicated in different disease states playing both protective and counter-protective roles. The current review presents HSP27 in multiple disease contexts: renal injury and fibrosis, cancer, neuro-degenerative and cardiovascular disease, highlighting its role as a potential biomarker and therapeutic target.

## **Review**

### **Heat shock protein 27: canonical roles in response to stress**

**Figure 1:**
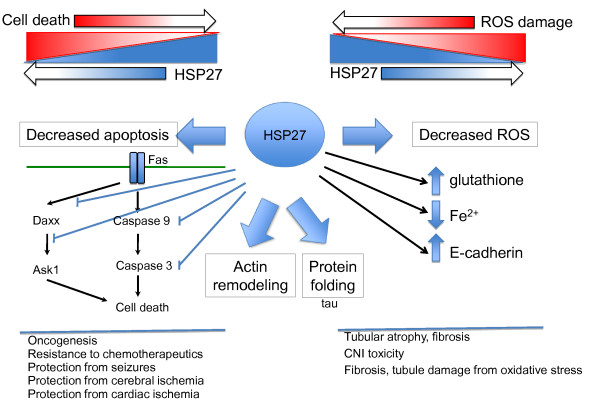
**Summary of some of the major mechanisms of HSP27 on disease states.** The primary mechanisms by which HSP27 acts are protein folding, effects on the actin cytoskeleton, reduction of oxidative stress and suppression of various modes of apoptosis or other kinds of cell death. The up-regulation that is a biomarker of some disease states is likely the cell’s attempt at rescue by using HSP27 to prevent cell death or to reduce the local oxidative stress. HSP27 presents in different oligomeric states, regulated by phosphorylation. While the phosphorylation state is important for some interactions, as discussed in the text, this has not been addressed in this figure.

Heat shock protein 27 (HSP27) belongs to the small molecular weight heat shock protein (HSP) family (12–43 kDa). HSP27 and other members of the small HSP family share a conserved c-terminal domain, the α-crystallin domain, which is identical to the vertebrate eye lens α-crystallin [1]. HSP27 was initially characterized in response to heat shock [2] as a protein chaperone that facilitates the proper refolding of damaged proteins [3,4]. Continued investigation of HSP27 revealed that the protein responds to cellular stress conditions other than heat shock; for example oxidative stress and chemical stress. During oxidative stress, HSP27 functions as an antioxidant, lowering the levels of reactive oxygen species (ROS) by raising levels of intracellular glutathione and lowering the levels of intracellular iron [5,6]. The protein functions as an anti-apoptotic agent under conditions of chemical stress by interacting with both mitochondrial dependent and independent pathways of apoptosis (Figure 1). HSP27 binds DAXX during Fas-FasL mediated apoptosis and prevents the subsequent binding of Ask1 by DAXX [7]. HSP27 also interacts with Bax and cytochrome c, thereby preventing mitochondrial dependent apoptosis [8,9]. HSP27 is particularly involved in protection from programmed cell death by inhibition of caspase-dependent apoptosis [10]. These anti-apoptotic properties in response to chemicals (perceived as stress by cells) has had major ramifications on the success of certain chemotherapies such as doxorubicin and gemcitabine [11,12]. Lastly, HSP27 has been characterized with the ability to regulate actin cytoskeletal dynamics during heat shock and other stress conditions, functioning both to promote actin polymerization and as an actin capping protein [13-15].

### **Functional regulation by phosphorylation**

HSP27 is present at basal levels in cells and tissues, and is organized as large oligomers [16]. The protein is phosphorylated by MAPKAP kinase 2/3 *via* the activation of the P38 MAPK pathway [14] at multiple serine residues (15, 78, and 82 in humans and 15 and 86 in rodent HSP25 [17,18]). Following phosphorylation, HSP27 reorganizes itself into smaller oligomers, often dimers and tetramers [4,6,7] and can interact with other proteins. HSP27 phosphorylation is dynamic and regulated by cellular conditions.

This simple change in phosphorylation state regulates many of the aforementioned canonical functions of HSP27. For example, small tetramers of phosphorylated HSP27 inhibit the upregulation of intracellular glutathione, thereby inhibiting its function as an antioxidant [4]. During Fas-FasL mediated apoptosis, it is phosphorylated HSP27 which binds DAXX thereby preventing DAXX from binding Ask1 and blocking the subsequent apoptosis cascade [7]. In the case of actin filament regulation, the phosphorylation state of HSP27 confers dual roles. Phosphorylated HSP27 prevents filament degeneration and promotes polymerization [14,15] while unphosphorylated HSP27 acts as an actin capping protein [13].

The phosphorylation of HSP27 also regulates its physical interaction with other proteins. In neutrophils, the serine/threonine kinase AKT is complexed with HSP27 and MAPKAP kinase 2, preventing constitutive neutrophil apoptosis and promoting an inflammatory response. Phosphorylated HSP27 dissociates from AKT, disrupting the signaling complex and promoting neutrophil apoptosis [19]. Conversely, in the study of atherosclerosis it was noted that phosphorylated HSP27 preferentially interacts with estrogen receptor β (ERβ), serving as a repressor and modulating estrogen signaling [20].

The functions of phosphorylated HSP27 are context specific. HSP27 phosphorylation has been utilized by researchers in order to develop a systematic understanding of HSP27 function under multiple experimental and disease conditions. Researchers have been able to mimic constitutive phosphorylation by substituting the phosphorylateable serine residues with aspartic acid [21] and have blocked phosphorylation by substituting phosphorylateable serine residues with alanine [22] or by replacing the serine coding region with codons for glycine [23]. Phosphorylated HSP27 is gaining prominence as a therapeutic target and biomarker of disease.

### **HSP27 and the kidney: potentials for diagnosis and therapy**

HSP27, and its rodent homolog HSP25, are up-regulated in various models of renal injury and fibrosis [24]. As researchers strive to elucidate the functional and therapeutic relevance of this observation, HSP27 is a strong candidate as a potential biomarker for renal disease.

#### ***Renal injury***

Renal injury frequently occurs as a consequence of the use of calcineurin inhibitor (CNI) therapy, an immunosuppressive regimen used during renal transplantation [25,26]. Cyclosporine A (CsA) is a commonly used CNI. CsA-mediated injury is characterized by severe nephrotoxicity, hypertension, and renal tubulointerstitial fibrosis. Prolonged CsA treatment resulted in the induction of HSP25 expression in the glomeruli and cortical tubules of rats [26]. The addition of melatonin resulted in amelioration of fibrosis and down-regulation of HSP25 and alpha-crystallin similar to untreated controls [26]. While the role of HSP25 in this experiment is unclear, it is possible that the antioxidant effects of both melatonin and HSP25 are similar and the presence of one eliminates the need for the other. In an experimental model of hypertension, Ishizaka *et al.* found that long-term angiotensin II treatment in rats induced HSP25 expression in the proximal tubular epithelial cells as well as the endothelial and medial smooth muscle cells of the renal artery [27]. This induction was dependent on angiotensin receptor II activation, rather than the level of hypertension. The authors speculated that there may be a potential protective role for HSP25/27 in kidneys with hypertensive nephropathy, similar to small molecular HSP32 (hemeoxygenase I; HO-I) which also has antioxidant properties [28]. In these experiments, HSP25/27 is protective, utilizing the antioxidant properties of this molecule.

Chronic allograft nephropathy (CAN) is a state of chronic stress following transplant which is characterized by chronic inflammation. Investigators have observed an induction of HSP25/27 and a shift in the expression pattern from the medulla of the kidney to the cortex [29]. This ‘re-localization’ was accompanied by markers of apoptosis such as Bax and FasL as well as markers of hypoxia such as HIF-1α and MnSOD. The authors determined that the induction of HSP25/27 expression levels and the change in the pattern of expression were hallmarks of the response of the allograft to CAN-related hypoxia and oxidative stress. Similar changes were also seen in patients with CAN. Using BOLD-MR technology the same investigators demonstrated impaired intra-renal oxygenation in CAN [30]. Serum HSP27 levels were significantly increased when compared to healthy volunteers. In addition, there was a strong correlation between intra-renal oxygenation and serum HSP27 levels [30]. More recent studies examining renal perfusion in kidney transplant recipients with magnetic resonance imaging demonstrated a correlation between serum HSP27 and kidney allograft perfusion, suggesting that HSP27 may be a viable biomarker of CAN-induced hypoxia and renal perfusion after transplantation [31]. In summary, in these studies HSP27 is again implicated in the context of oxidative stress. When conditions in the kidney become hypoxic, HSP27 is up-regulated as a protective response.

The kidney is susceptible to ischemic injury, which is characterized in part by renal epithelial cell injury and also the up-regulation of heat shock proteins [32,33]. Increased HSP25 expression was observed in the cortex and medulla of the kidney, along with decreased glomerular phosphorylation following ischemia [33]. These reports suggested a protective role for HSP25/27 as an actin cytoskeletal remodeling protein, both to maintain tubular integrity during injury and also to anchor the sodium potassium ATPase to the actin cytoskeleton [33,34]. A similar role of maintaining actin cytoskeletal integrity was attributed to the up-regulation of HSP27 during injury to the podocytes of the glomerulus and during the ensuing glomerulonephritis [35].

In order to closely examine the potential therapeutic properties of HSP27 during ischemic injury, Chen *et al.*[36] developed a transgenic mouse model that globally overexpressed HSP27. *In vitro* experiments using primary cultures of renal epithelial cells from the transgenic mice resisted ischemic injury upon peroxide induced necrosis. However the transgenic mice themselves fared worse than wild-type control mice, with decreased renal function and increased inflammation upon ischemia-reperfusion. These experiments highlight the need for kidney specific HSP27 expression in order to properly explore the therapeutic nature of HSP27. A follow up study by the same group introduced HSP27-lentiviral constructs *via* injections into the kidneys 2 days prior to induction of ischemia [37]. In this study, HSP27 overexpressing mice demonstrated significantly lower apoptosis and necrosis, as well as lower induction of mRNAs of various pro-inflammatory cytokines. These mice also demonstrated better F-actin preservation in the proximal tubules, thus substantiating a therapeutic role for HSP27 as an actin remodeling protein during conditions of ischemic injury. Summarizing, these experiments highlight the third major function of HSP25/27, cytoskeletal remodeling.

#### ***Renal fibrosis***

Renal tubulointerstitial fibrosis is a final point of pathological confluence for a variety of kidney diseases and injuries. It has often been defined as the final common pathway leading to renal failure during the progression of kidney diseases of varied etiology [38-40].

Uretero-pelvic junction (UPJ) obstruction (occlusions in the urinary tract, at the base of the kidney) and its experimental counterpart, unilateral ureteral obstructions (UUO, surgical obstruction at the uretero-pelvic junction) are common models for the study of renal tubulointerstitial fibrosis. An in-depth study by Valles *et al*. [41] examined HSP27 expression in 22 patients with UPJ obstructions (congenital obstructions for 2.1+/−0.41 years). The authors attempted to correlate kidney function and duration of obstruction to HSP27 (and HSP70) expression. They found that UPJ obstructions were characterized by tubulointerstitial fibrosis and oxidative stress. Their study concluded that patterns of HSP27 expression correlated with the duration of obstruction and that HSP27 was induced as an adaptive response during UPJ obstructions.

**Figure 2:**
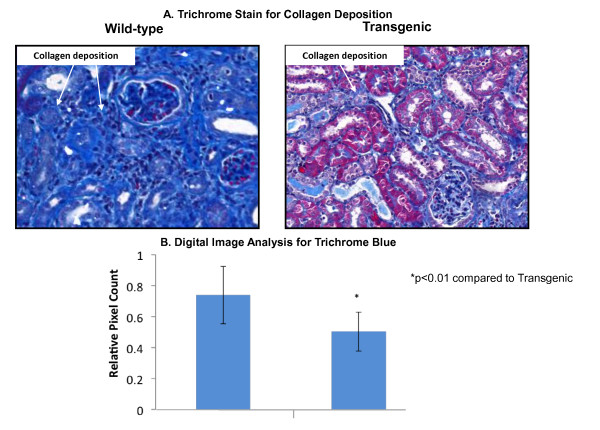
**HSP27 overexpression was associated with decreased fibrosis after UUO.** Tissue sections prepared from 14 day UUO kidneys from wild type and KAP2-HSP27 transgenic mice were stained with trichrome aniline blue and quantitatively analyzed using the Nuance digital analysis software system. Trichrome blue staining was significantly reduced in the transgenic obstructed mice as compared to wild-type mice (*P* < 0.01). The data shown are representative of the group averages (*n* = 3 in each group).

Tubular atrophy is one of the defining features of renal tubulointerstitial fibrosis. The loss of tubular function and integrity occurs primarily *via* the apoptosis of renal tubular epithelial cells [42]. Loss of E-cadherin at the cell membrane is a common feature of renal tubulointerstitial fibrosis. Following the observation of HSP27 induction *in vivo* during UUO, Vidyasagar *et al.*[24] examined the potential protective effects of HSP27 *in vitro* in TGF-β1 treated proximal tubular epithelial cells, NRK52E. HSP27 was overexpressed in NRK52E cells by transiently transfecting with a plasmid-HSP27 cDNA construct. TGF-β1 treatment alone resulted in diminished E-cadherin protein levels. HSP27 overexpression in TGF-β1 treated cells resulted in E-cadherin protein levels which were comparable to untreated and untransfected controls. In addition, the E-cadherin transcriptional repressor, Snail was also down-regulated. These experiments suggested a therapeutic role for HSP27 delaying tubular injury by maintaining E-cadherin protein levels, possibly through the down-regulation of Snail. More recent data using mice that overexpress HSP27 in the kidney show that during UUO, the transgenic mice have significantly less fibrosis than wild type (Figure 2).

Both UPJ/UUO and TGF-β1 treatment result in renal tubulointerstitial fibrosis. In both cases, HSP27 appears to play an adaptive or protective role, ameliorating the fibrotic processes that characterize kidney disease.

### **HSP27: a biomarker of disease and emerging therapeutic target**

#### ***Cancer***

Increased transcription of HSPs in tumor cells is due to the loss of p53 functions as well as to higher expression of proto-oncogenes such as *HER* and *c-Myc* and is crucial to tumorigenesis [10]. The potent cytoprotective and folding properties of HSPs are co-opted during oncogenesis, as the HSPs become expressed at high levels to enable tumor cell growth and survival. HSP27 is particularly involved in protection from programmed cell death by inhibition of caspase-dependent apoptosis.

Heat shock proteins, especially HSP27, are associated with poor prognosis and treatment in many types of cancer including gastric, liver, and prostate carcinoma, osteosarcoma, rectal, lung, and breast cancer [43-45]. HSP27 is also implicated in resistance to chemotherapy in breast cancer [44] and leukemia [43] and is associated with acquisition of drug-resistant phenotypes [10].

On the positive side, HSPs can also become targets for cancer therapy drugs, as well as targets for the immune system. The massive release of HSP due to widespread tumor cell necrosis after cytotoxic drugs can lead to CD8+ T cell mediated anti-tumor immune responses [46].

Langer *et al*. [47] compared protein expression profiles in patients with esophageal adenocarcinomas who were responsive and non-responsive to neoadjuvant platin/5-fluorouracil based chemotherapy. Contrary to findings in breast cancer, the study concluded that low HSP27 expression correlated with non-responsiveness to the chemotherapy regimen. This is one of the few instances in which low levels of HSP27 expression correlates with a negative outcome in cancer.

Tweedle *et al.* conducted a study that aimed to characterize HSP27 levels in patients diagnosed with colon or rectal cancer. They found a highly significant association between high HSP27 expression and incomplete resection margins in rectal cancer. Elevated HSP27 was also associated with poor survival. When analyzed separately, HSP27 expression was not associated with survival in the colon cancer group, but was strongly correlated to poor survival in the rectal cancer group [45].

Metastatic breast cancers that overexpress *Her2* (epidermal growth factor receptor (EGFR) related tyrosine kinase) are treated with Herceptin, a monoclonal antibody. However, Herceptin resistance can ensue, reducing the efficacy of Herceptin-based chemotherapies. A 2008 study by Kang *et al*. [44] found that metastatic breast cancer cell lines that overexpress Her2 and that are resistant to Herceptin (SK-BR3 HR), also overexpress HSP27. When the authors down-regulated HSP27 protein levels by transfecting with siRNA, Herceptin resistance was greatly reduced in SK-BR3-HR cells. The study also found that HSP27 could form a complex with Her2, suggesting a potential mechanism by which the protein potentiates Herceptin resistance. An earlier study found that *Her2* overexpressing breast cancer tumors showed increased expression of phosphorylated HSP27, particularly at serine 78 [44,48].

The association of HSP27 with tumor-specific antigens leads to a local antibody response to HSP27. The presence of IgA anti-HSP27 antibodies has also emerged as a diagnostic marker for gynecological malignancies such as ovarian, endometrial, and cervical cancer. Neither patients with benign gynecological cancers nor normal patients demonstrate the presence of IgA anti-HSP27 antibodies. In addition, anti-cancer regimens also lead to the decrease in IgA anti-HSP27 antibodies [49].

Preliminary studies exist, targeting HSP27 in cancer therapy primarily through the down-regulation/inhibition of HSP27 either by using chemical inhibitors or by using anti-sense oligonucleotides. Hsu *et al.* showed that while traditional chemotherapeutic agents were able to modestly reduce tumor volume, adding the HSP27 inhibitor quecertin resulted in a significant reduction of tumor volumes *in vivo*[50]. Thus, pharmacological inactivation of HSP27 sensitized A549 lung cancer stem cells to apoptotic cell death *in vitro* and of tumors in an *in vivo* mouse model of lung cancer [50]. The use of anti-sense oligo-nucleotides has moved into clinical trials with OGX-427, an anti-sense oligo-nucleotide that is complementary to HSP27. Currently, OGX-427 is in phase II clinical trials in the United States and Canada for various cancers such as lung, ovarian, breast, and pancreatic cancer [51].

In summary, the cytoprotective functions exhibited by HSP27 may have a protective role in kidney fibrosis. However, overexpression of HSP27 is associated with poorer outcomes in cancer by protecting malignant cells from undergoing apoptosis. As such, HSP27 could become a target for the treatment and prevention of both fibrosis and cancer.

#### ***Neuro-degenerative disease and neuronal injury***

Neuro-degenerative diseases are characterized by the accumulation of mis-folded proteins. In their 2010 study Abisambra *et al.*[21] not only highlighted the potential therapeutic properties of HSP27 but also the variability in function between *in vitro* and *in vivo* models, and the importance of dynamic cycling between phosphorylated and unphosphorylated forms. *In vitro,* HSP27 and its constitutively phosphorylated mutant form both interacted with and prevented tau accumulation. However, upon adenoviral delivery to tau transgenic mice, only the wild-type form of HSP27 was able to prevent tau filament accumulation. Constitutively phosphorylated HSP27 increased tau accumulation, highlighting the importance of phosphorylation state regulation and dynamic shuttling between the two states.

Cerebral ischemia causes neuronal injury and is characterized by cell death. Kato *et al*. [52] demonstrated the increased expression of HSP27 following ischemia and reperfusion, in a time-dependent manner, in surviving microglia and astrocytes. In order to determine the function of HSP27 up-regulation in cerebral ischemia, Stetler *et al*. [53] designed transgenic mice that globally overexpressed HSP27. The study determined that HSP27 inhibited ASK1-dependent MKK4/JNK activation, upstream of the mitochondrial dependent pathways of apoptosis. Thus HSP27 overexpression has potential protective effects during cerebral ischemia and subsequent neuronal injury, implicating it as a potential therapeutic agent during stroke.

Akbar *et al.*[54] demonstrated lower seizure activity in response to kainic acid in transgenic mice overexpressing HSP27 in the brain and spinal cord. In keeping with the anti-apoptotic functions of HSP27 during neuronal injury, these transgenic mice also showed reduced apoptosis and caspase 3 induction. This study underscores the potential neuroprotective effects of HSP27 under conditions of neurotoxicity.

In summary, HSP27 protects from neuronal injury primarily through its role as an anti apoptotic agent.

#### ***Cardiovascular disease***

Lastly, HSP27 has been implicated in cardiovascular disease both as a potential biomarker of disease and injury as well as a potential therapeutic target [55]. As with ischemic brain injury, HSP27 overexpression protects against ischemic injury of cardiac myocytes, irrespective of phosphorylation state [22].

Atherosclerosis is a chronic multifactorial disease that is characterized by the presence of lipids and extracellular matrix material as plaques in the arteries [55]. Martin-Ventura *et al.*[56] identified HSP27 as a potential biomarker of atherosclerosis amongst a cohort of differentially secreted proteins. They found HSP27 expression to be decreased in atherosclerotic plaques with normal expression in healthy arteries. Wick *et al.*[57] confirmed that during atherosclerosis, HSP27 is down-regulated in the most severe plaques or the plaque core, while normal adjacent tissue expressed higher levels of HSP27. An in-depth review of the involvement of HSP27 in various cardiac diseases, Ghayour-Mobarhan *et al*. [55] suggested that reduced HSP27 expression may favor smooth muscle growth and plaque formation. As a therapeutic intervention, maintaining normal HSP27 levels in plaques may prevent plaque formation.

Though conflicting data exist as to the validity of HSP27 antibody titers as a biomarker of cardiovascular diseases, Shams *et al.* suggest that HSP27 serum antibody titers are directly associated with the severity of chest pain [58].

In cardiovascular disease, HSP27 overexpression protects against ischemic injury (presumably utilizing its antioxidant properties) and also acts as a biomarker of disease.

## **Conclusions**

The present summary highlights some of the recent data examining HSP27 in its diagnostic and therapeutic capacities. Despite increasing evidence to substantiate HSP27 as biomarker in many disease states, more studies are needed to address the discrepancies and evaluate the specific response of this small HSP depending on the context. Another challenge lies in utilizing this knowledge towards therapy. Organ specific targeting is necessary and a thorough understanding of HSP27 phosphorylation state in each disease condition is essential. The precedent has been set by cancer research to utilize HSP27 as a therapeutic target. The future of HSP27 therefore augurs its development as a multidimensional therapeutic agent and target.

## **Abbreviations**

BOLD-MR, Blood oxygen level dependent magnetic resonance; CAN, Chronic allograft nephropathy; CNI, Calcineurin inhibitor; CsA, Cyclosporine A; HSP, Heat shock protein; ROS, Reactive oxygen species; UPJ, Uretero-pelvic junction obstruction; UUO, Unilateral ureteral obstruction.

## **Competing interests**

Authors have no competing interests.

## **Authors’ contributions**

AV did most of the research and writing, AD conceived the original concept for the review, NAW assisted with research and editing. All authors have read and approved the final manuscript.

## Source of Funding

NIDDK-DK067981-5 
